# FADD promotes type I interferon production to suppress porcine reproductive and respiratory syndrome virus infection

**DOI:** 10.3389/fvets.2024.1380144

**Published:** 2024-04-08

**Authors:** Xiaobo Chang, Mengqi Wang, Zhaopeng Li, Lei Wang, Gaiping Zhang, Yafei Chang, Jianhe Hu

**Affiliations:** ^1^Postdoctoral Innovation Practice Base, College of Animal Science and Veterinary Medicine, Henan Institute of Science and Technology, Xinxiang, China; ^2^College of Animal Science and Veterinary Medicine, Henan Institute of Science and Technology, Xinxiang, China; ^3^College of Veterinary Medicine, Henan Agricultural University, Zhengzhou, China

**Keywords:** PRRSV, FADD, MAVS, type I IFN signaling, antiviral response

## Abstract

Porcine reproductive and respiratory syndrome (PRRS) is an epidemic animal infectious disease worldwide, causing huge economic losses to the global swine industry. Fas-associated death domain (FADD) was previously reported to be an adaptor protein that functions in transferring the apoptotic signals regulated by the death receptors. In the current study, we unravel its unidentified role in promoting type I interferon (IFN) production during PRRS virus (PRRSV) infection. We identified that FADD inhibited PRRSV infection via promotion of type I IFN transcription. Overexpression of FADD suppressed the replication of PRRSV, while knockout of FADD increased viral titer and nucleocapsid protein expression. Mechanistically, FADD promoted mitochondrial antiviral signaling protein (MAVS)-mediated production of IFN-β and some IFN-stimulated genes (ISGs). Furthermore, FADD exerted anti-PRRSV effects in a MAVS-dependent manner and increased the type I IFN signaling during PRRSV infection. This study highlights the importance of FADD in PRRSV replication, which may have implications for the future control of PRRS.

## Introduction

Porcine reproductive and respiratory syndrome (PRRS), an important epidemic disease, characterized by late term gestation reproductive failure in sows and general respiratory symptoms in pigs of all ages and sexes, causing tremendous amounts of economic losses to the swine industry (1–4). PRRSV, the causative agent of PRRS, is an enveloped virus with 15 kb positive-strand RNA genome containing at least 10 open reading frames (ORFs), belonging to the Arteriviridae family, Nidovirales order (5–7). PRRSV is divided into PRRSV-1 and PRRSV-2, and PRRSV-2 strains are predominantly prevalent in China (8). In addition, PRRSV infection can trigger host innate immune response (9).

Type I IFNs (IFN-α/β) play pivotal roles in antiviral response. Upon virus infection, viral nucleic acids as pathogen-associated molecular patterns (PAMPs) can be detected by the cellular receptors such as RNA sensor retinoic acid-inducible gene I (RIG-I)-like receptors (RLRs), which recruits the mitochondrial antiviral signaling (MAVS), leading to TBK1-dependent phosphorylation of IRF3, and transcription of type I IFNs and ISGs (10, 11). Moreover, type I IFNs and numerous ISGs have been confirmed to have antiviral effects against PRRSV (12–15).

FADD, initially described as a key adaptor protein, interacted with the death receptors (DRs), such as FAS and the tumor necrosis factor receptor 1 (TNF-R1), leading to the recruitment and the activation of procaspase-8, and consequent apoptosis of the cell (16, 17). In recent years, however, FADD has been reported to be involved in a variety of non-apoptotic processes, including cell cycle progression, embryonic development, hematopoiesis and lymphocyte cell cycle progression and survival (18, 19). Besides, FADD has emerged as an actor of innate immunity and inflammation. In particular, FADD and RIPK1 could form the innateosome in response to viral RNA recognized by intracellular receptors, thereby regulating TBK1-mediated activation of IRF3 and IFN-β production (17, 20). And several studies have shown that the replication of RNA viruses, such as VSV, ZIKV, influenza virus and encephalomyocarditis virus (EMCV), is greatly facilitated in FADD deficient human and mouse fibroblasts, which highlights the importance of FADD in the antiviral immune response (21–24). However, the roles and mechanism of FADD in response to PRRSV have not been elucidated.

In this study, we found that FADD overexpression restrained the replication of PRRSV, whereas FADD silence promoted PRRSV replication. Besides, FADD positively regulated MAVS-mediated type I IFN signaling pathway. More importantly, MAVS was essential for FADD to repress PRRSV replication, and the antiviral activity of FADD depended on the transcription of type I IFN. These findings may contribute to understanding the cellular proteins in the role of regulating PRRSV replication.

## Materials and methods

### Cells and virus

MARC-145 (a PRRSV permissive cell line subcloned from MA-104) cells and HEK-293T cells were cultured in Dulbecco’s modified Eagle’s medium (DMEM, Solarbio) supplemented with 10% fetal bovine serum (FBS, Gibco), 100 U/mL penicillin (Gibco) and 100 μg/mL streptomycin (Gibco) at 37°C in a humidified atmosphere of 5% CO_2_. PRRSV strain BJ-4 (GenBank No. AF331831) stored in Henan Provincial Key Laboratory of Animal Immunology was a kind gift from Prof. Hanchun Yang (China Agricultural University).

### Antibodies and reagents

Rabbit anti-FADD antibody and Rabbit anti-MAVS antibody were purchased from Proteintech. Mouse anti-Flag M2 monoclonal antibody, mouse anti-c-Myc monoclonal antibody were purchased from Sigma-Aldrich. PRRSV N protein antibody was purchased from GeneTex. Mouse anti-β-actin monoclonal antibody was purchased from GenScript. The secondary antibodies conjugated to HRP were purchased from Proteintech. Polyinosinic-polycytidylic acid (polyI:C) was purchased from Sigma-Aldrich. Lipofectamine 2000 transfection reagent and Lipofectamine RNAiMAX transfection reagent were purchased from Invitrogen.

### Construction of plasmids

The cDNA of FADD was cloned into pCMV-Myc, named as FADD-Myc. The genes of RIG-I, MDA5, MAVS, TBK1 and IRF3 were cloned into pCMV-Flag, which were termed as RIG-I-Flag, MDA5-Flag, MAVS-Flag, TBK1-Flag and IRF3-Flag, respectively. The IFN-β luciferase reporter plasmid (IFN-β-Luc), has been constructed as previously described (25). The pRL-TK (Promega), containing a Renilla-luciferase reporter gene, was used as an endogenous control in the dual luciferase reporter assay system. All primers used are listed in Table 1.

**Table 1:** Primers used for expression plasmid construction.

Primers	Sequences (5′ to 3′)
FADD-F	CCCAAGCTTATGCGCGGTTCCCGGAGTT
FADD-R	CCGCTCGAGTCAGGATGCTTCGGAGGTGG
RIG-I-F	TCCCCCCGGGCATGACTACGGAGCAGCGGCGCAGCCT
RIG-I-R	ATCTGTCGACTCATTTGGCCATTTCTGCTGGAT
MDA5-F	CGCGGATCCATGTCGAATGGGTATTCCACAGAC
MDA5-R	CCGCTCGAGCTAATCCTCATCACTAAATAAACAGCA
MAVS-F	CCCAAGCTTATGCCGTTTGCTGAAGACAAGACCT
MAVS-R	CCGCTCGAGCTAGTGCAGGCGCCGCCGGTACATC
TBK1-F	CGCGGATCCATGCAGAGCACTTCTAATCATCT
TBK1-R	CCGCTCGAGCTAAAGACAGTCAACGTTGCGA
IRF3-F	CCCAAGCTTATGGGAACCCCAAAGCCACGGAT
IRF3-R	CCGCTCGAGTCAGGTCTCCCCAGGGCCCTGGAA

### RNA interference

Small interfering RNAs (siRNA) targeting FADD (siFADD: 5′-GGAAGAACGCAGAGAAGAATT-3′), MAVS (siMAVS: 5′-GCAUCUCUUCAGUACCCUUTT-3′) and the non-target control siRNA (siNC: 5′-UUCUCCGAACGUGUCACGUTT-3′) were designed and synthesized by GenePharma. MARC-145 cells were transfected with 50 nM siRNA using Lipofectamine RNAiMAX transfection reagent. Twenty-four hours later, the cells were infected with PRRSV at a multiplicity of infection (MOI) of 0.1. And then, the cells or the supernatants were harvested at the indicated times (24, 36 and 48 h) for subsequent experiments.

### Quantitative real-time PCR analysis

Total RNAs were extracted using Cell/Tissue Total RNA Isolation Kit (Vazyme), and the reverse transcription cDNAs were prepared from total RNAs using the HiScript^®^ III All-in-one RT SuperMix Perfect for qPCR (Vazyme). The cDNAs were amplified by quantitative real-time PCR (qPCR) using ChamQ Universal SYBR qPCR Master Mix (Vazyme). The relative mRNA level of gene expression was evaluated using the 2^−ΔΔCT^ method with glyceraldehyde-3-phosphate dehydrogenase (GAPDH) mRNA as an endogenous control. The primers for qPCR are listed in Table 2.

**Table 2:** Primers used for qPCR.

Primers	Forward primer (5′ to 3′)	Reverse primer (5′ to 3′)
ORF7	AAACCAGTCCAGAGGCAAGG	GCAAACTAAACTCCACAGTGTAA
FADD	GACCTCTTCTCCGTACTGCTGG	TAAATGCTGCACACAGGTCTTCTTC
IFN-β	ACGGCTCTTTCCATGAGCTAC	GTCAATGCAGCGTCCTCCTT
ISG15	CTGAAGGCAAAGATCGCCCA	GTCGTTCCTCACCAGGATGC
ISG56	AGGAAACACCCACTTCGGTC	CCTCTAGGCTGCCCTTTTGT
OAS1	TGGTGGAGACACAAAGGGTT	AACCATGTTGCTGATACATCCTG
MAVS	CTGCCTCACAGCAAGAGACCA	GTAGACACAGGCCACTTCGTC
GAPDH	GAAGGTGAAGGTCGGAGTCA	CATGTAAACCATGTAGTTGAGGTC

### Dual-luciferase reporter assays

Luciferase assays were conducted by the Dual-Luciferase^®^ Reporter Assay System according to Promega’s instructions. In brief, MARC-145 cells or HEK-293T cells were transfected with indicated expression plasmids, IFN-β luciferase reporter plasmid and pRL-TK renilla luciferase reporter plasmid by using Lipofectamine 2000 transfection reagent (Invitrogen). At 36 hpt, the cells were transfected with polyI:C (10 μg/mL) for 12 h. PolyI:C, the synthetic analog of double-stranded RNA (dsRNA), is experimentally used to trigger type I IFN production (26). Then, the transfected cells were lysed in passive lysis buffer and subjected to luciferase activity measurement using dual-luciferase reporter assay system (Promega).

### Western blot analysis

The treated or mock MARC-145 cells were harvested and washed three times with cold PBS, and then lysed in RIPA Buffer (Sigma-Aldrich) containing protease inhibitor cocktail (Roche). Protein concentration was determined using Micro BCA protein assay kit (Thermo Fisher Scientific). Equal amounts of protein lysates with SDS-PAGE loading buffer were run on 10% SDS-PAGE gels and transferred to 0.22 μm PVDF membranes. Then membranes were blocked in 5% skim milk and incubated with the indicated primary antibody overnight at 4°C, followed by the incubation with HRP-conjugated secondary antibodies for 2 h at room temperature. Subsequently, Luminescent signals were detected with SuperSignal^™^ chemiluminescent HRP substrates (Thermo Fisher Scientific). The relative levels of target proteins normalized to β-actin were analyzed using Image J software.

### Virus titration

Virus titers were determined according to a previous report (27). Briefly, MARC-145 cells, grown in 96-well plates, were infected with 10-fold serial dilution of samples. After 1 h incubation at 37°C, the supernatants were replaced with fresh DMEM containing 2% FBS. Five days post infection, the cytopathic effect (CPE) characterized by clumping and shrinkage of cells was visible in MARC-145 cells and the viral titers were calculated by 50% tissue culture infective dose (TCID_50_) according to the method of Reed-Muench (28).

### Statistical analysis

All experiments were repeated independently at least three times. The experimental data were presented as group mean and standard deviation (SD) and analyzed by the unpaired two-tailed student *t*-test with GraphPad. The asterisks in the figures indicate significant differences (^*^p < 0.05, ^**^p < 0.01, and ^***^p < 0.001).

## Results

### FADD overexpression suppresses PRRSV replication

**Figure 1:**
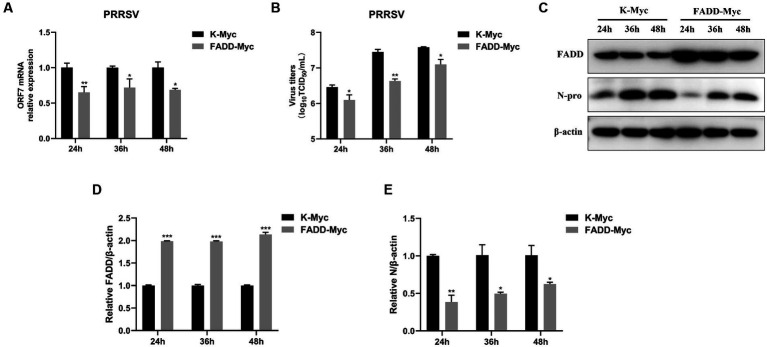
Overexpression of FADD inhibits PRRSV replication. MARC-145 cells were transfected with FADD-Myc or control vector for 24 h, and then infected with PRRSV at a MOI of 0.1 for another 24, 36 and 48 h. **(A)** The RNA levels of PRRSV were assessed by using qPCR. **(B)** The viral titers in cell culture supernatant were assayed and presented by TCID_50_. **(C)** The expression levels of FADD and N protein of PRRSV were analyzed using western blotting. **(D,E)** The relative intensity ratios of FADD and N protein were also shown in this figure using Image J software. All experiments were repeated at least three times with similar results. ^*^p < 0.05, ^**^p < 0.01, and ^***^p < 0.001. The statistical significance of differences was determined using student’s *t*-test.

In the previous study, we reported that FADD is a target of miR-382-5p increased during PRRSV infection (29). To verify the biological significance of FADD during PRRSV infection, we examined the effect of FADD on PRRSV replication. FADD was overexpressed in the MARC-145 cells by transfection with FADD-Myc. As shown in Figure 1, when FADD was overexpressed, the mRNA levels of PRRSV N gene were lower than those in control cells at 24–48 h (Figure 1A). Subsequently, we measured the viral titers from the supernatants of FADD overexpressed or control cells. Overexpression of FADD reduced PRRSV release as shown by TCID_50_ assay (Figure 1B). Furthermore, FADD overexpression markedly decreased the abundance of PRRSV N protein (Figures 1C–E), which indicated that FADD overexpression showed inhibitory effects on PRRSV.

### FADD knockdown promotes PRRSV infection

**Figure 2:**
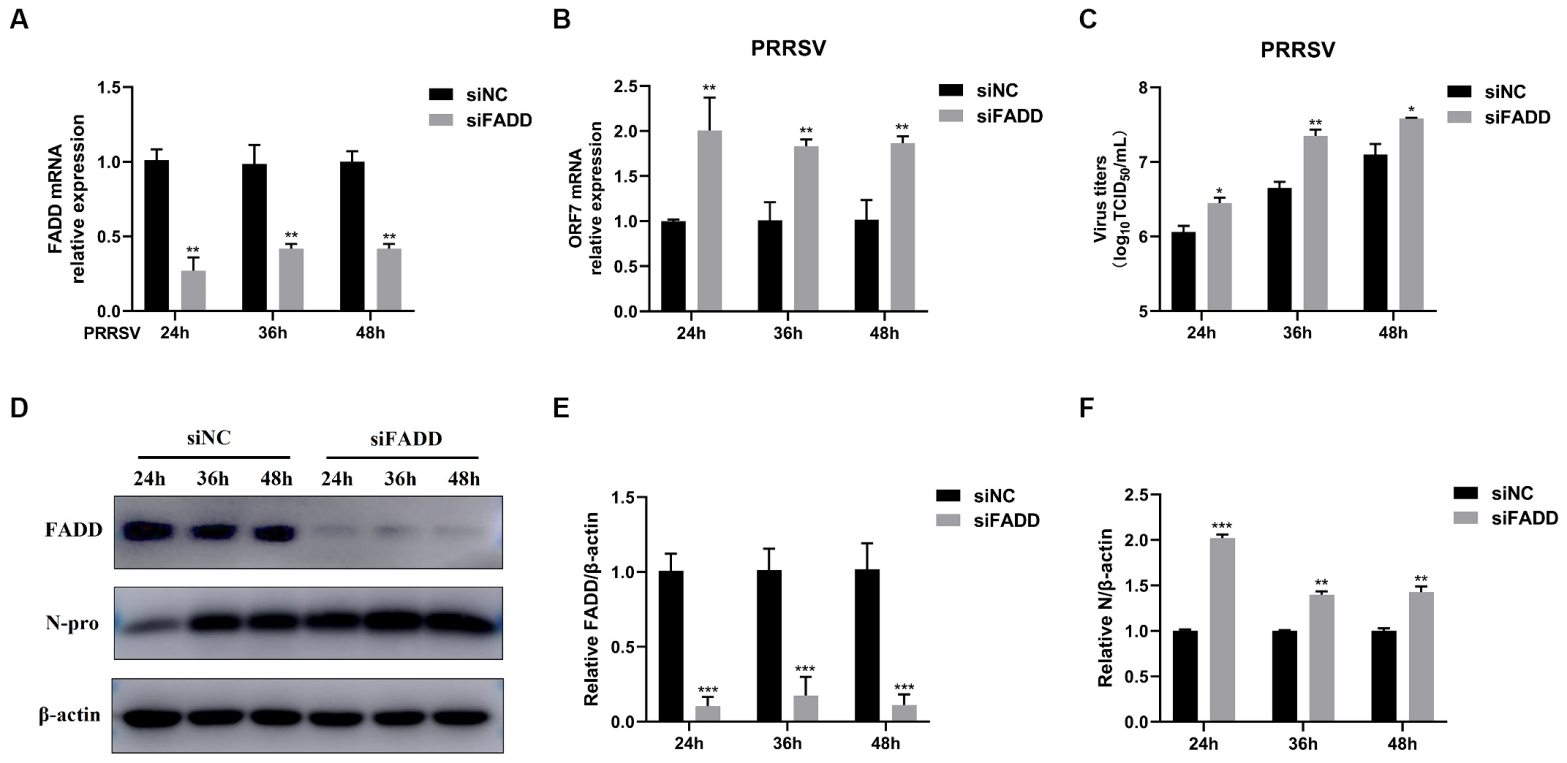
FADD knockdown promotes PRRSV infection. MARC-145 cells were transfected with siFADD or a nontargeting control (siNC) for 24 h, then the cells transfected with siFADD or siNC were infected with PRRSV at a MOI of 0.1 for 24, 36, 48 h. **(A)** The knockdown efficiency of FADD and **(B)** the mRNA levels of PRRSV ORF7 were examined by qPCR. **(C)** The virus titers were analyzed by TCID_50_. **(D)** The expression levels of N protein were detected by western blotting with a rabbit anti-PRRSV N protein polyclonal antibody. **(E,F)** The relative expression levels of FADD and N protein were analyzed by Image J software. The results are representative of three independent experiments (the means ± SD). ^*^p < 0.05, ^**^p < 0.01, and ^***^p < 0.001. The statistical significance of differences was determined using student’s *t*-test.

In contrast, we examined the effects of FADD knockdown on PRRSV infection. We used specific siRNA to down-regulate the expression of endogenous FADD, which could efficiently reduce the FADD expression (Figures 2A,D,E). Then MARC-145 cells transfected with siRNAs were infected with PRRSV for 24, 36 and 48 h, compared with nontargeting control siRNA (siNC)-transfected cells, PRRSV RNA levels and viral titers in FADD knockdown cells increased markedly at 24, 36 and 48 hpi (Figures 2B,C). Similarly, silencing of FADD significantly enhanced the expression levels of N protein (Figures 2D–F). These data suggest FADD is involved in PRRSV infection and is a cellular antiviral factor against PRRSV.

### FADD promotes type I IFN production following polyI:C stimulation

**Figure 3:**
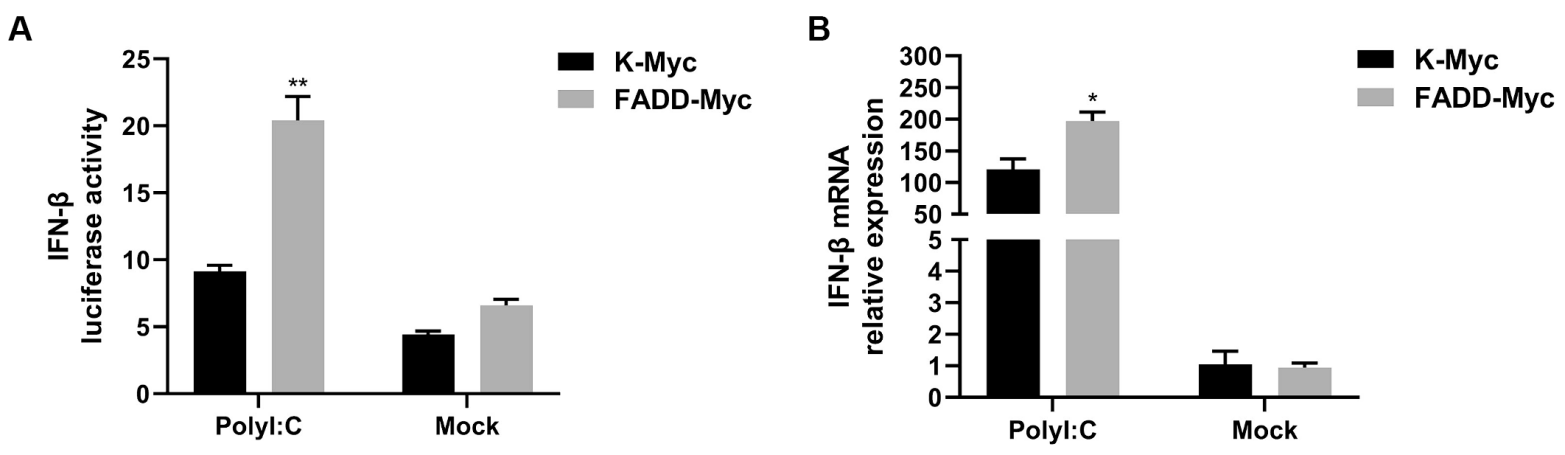
FADD promotes ployI:C-mediated production of IFN-β. **(A)** HEK-293T cells were transfected with IFN-β reporter plasmid and FADD-Myc or control vector, and 36 h later, the cells were transfected with polyI:C (10 μg/mL) for 12 h, and then the luciferase activities of IFN-β were assayed by dual-luciferase report assay. **(B)** MARC-145 cells were transfected with FADD-Myc or control vector for 36 h, then transfected with polyI:C for 12 h, the mRNA levels of IFN-β were measured by qPCR. The results are representative of three independent experiments (the means ± SD). ^*^p < 0.05 and ^**^p < 0.01. The statistical significance of differences was determined using student’s *t*-test.

FADD and RIPK1 could form an innateosome in response to viral dsRNA and then regulate TBK1-mediated IFN-β production (17). So next we wonder to know whether FADD could regulate polyI:C-mediated type I IFN production. Poly (I:C), the synthetic analog of double-stranded RNA (dsRNA), is experimentally used to trigger type I IFN production (30). The results showed that FADD overexpression in MARC-145 cells increases the IFN-β promoter activity (Figure 3A) and the transcriptional levels of IFN-β triggered by polyI:C (Figure 3B). These data suggest that FADD participates in promoting type I IFN signaling.

### FADD promotes MAVS-mediated type I IFN signaling

**Figure 4:**
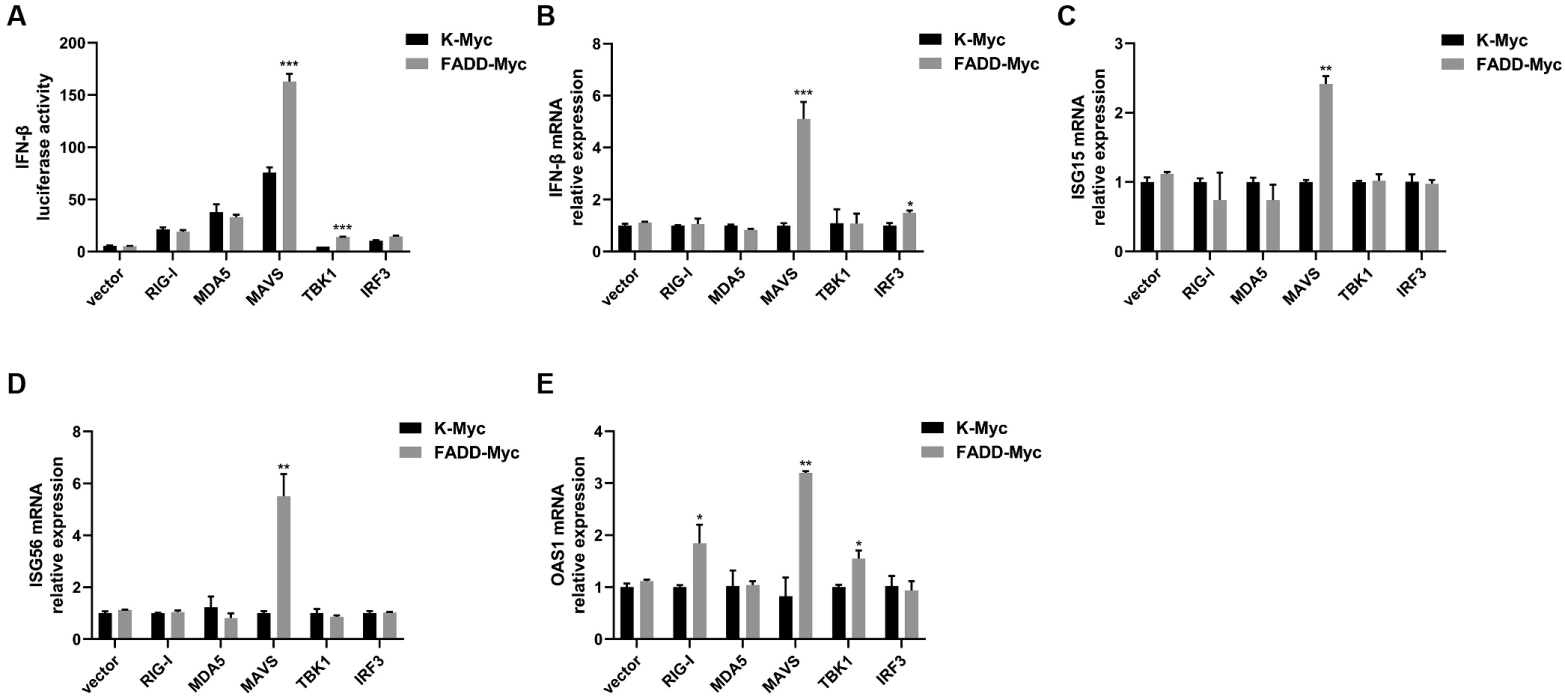
FADD promotes MAVS-mediated type I IFN signaling. **(A)** HEK-293T cells were transfected with IFN-β reporter plasmid, FADD-Myc, and RIG-I, MDA5, MAVS, TBK1, or IRF3 plasmids. Luciferase assays were performed at 48 h. **(B–E)** MARC-145 cells were transfected with FADD-Myc and RIG-I, MDA5, MAVS, TBK1, or IRF3 plasmids. And 48 h later, the transcriptional levels of IFN-β **(B)**, ISG15 **(C)**, ISG56 **(D)** and OAS1 **(E)** were detected by qPCR. The results are representative of three independent experiments (the means ± SD). ^*^p < 0.05, ^**^p < 0.01, and ^***^p < 0.001. The statistical significance of differences was determined using student’s *t*-test.

**Figure 5:**
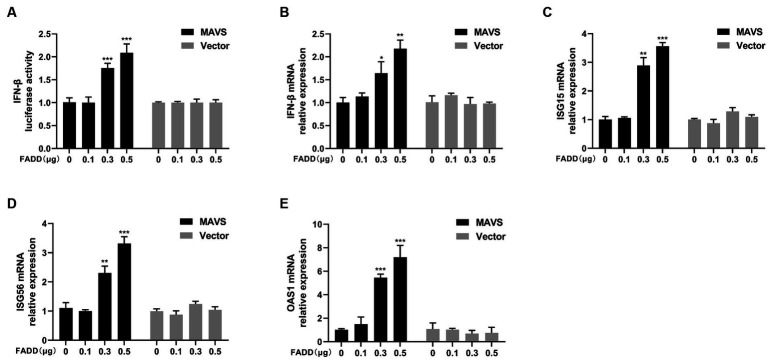
FADD promotes MAVS-mediated type I IFN signaling in a dose-dependent manner. **(A)** HEK-293T cells were transfected with MAVS, increasing amounts of FADD-Myc and IFN-β reporter plasmid, at 48 hpt, the luciferase activities were analyzed using a dual-luciferase reporter assay. **(B–E)** MARC-145 cells were transfected with MAVS and increasing amounts of FADD-Myc, the mRNA levels of IFN-β **(B)**, ISG15 **(C)**, ISG56 **(D)** and OAS1 **(E)** were detected by qPCR. The results are representative of three independent experiments (the means ± SD). ^*^p < 0.05, ^**^p < 0.01, and ^***^p < 0.001. The statistical significance of differences was determined using student’s *t*-test.

Given that FADD governs polyI:C-triggered activation of IFN-β, we investigated the role and mechanism of FADD in the RLR-MAVS signaling pathway. HEK-293T cells were co-transfected with plasmids encoding FADD, components of the RIG-I pathway, and IFN-β reporter plasmid. The results showed that FADD greatly enhanced the IFN-β promoter activity induced by MAVS and TBK1 (Figure 4A). And the mRNA transcriptional levels of IFN-β were dramatically increased in FADD and MAVS co-transfected cells, mildly increased in FADD and IRF3 co-transfected cells (Figure 4B). Meanwhile, FADD also upregulated MAVS-induced the mRNA expressions of ISG15, ISG56 and OAS1 (Figures 4C–E). Besides, the mRNA expressions of OAS1 were also increased by RIG-I and TBK1 in FADD-transfected cells (Figure 4E). Subsequently, cells were co-transfected with MAVS, IFN-β reporter plasmid, and different concentrations of FADD, and we found that FADD increased MAVS-induced IFN-β promoter activity (Figure 5A) and the mRNA transcription levels of IFN-β, ISG15, ISG56 and OAS1 in a dose-dependent manner (Figures 5B–E).

**Figure 6:**
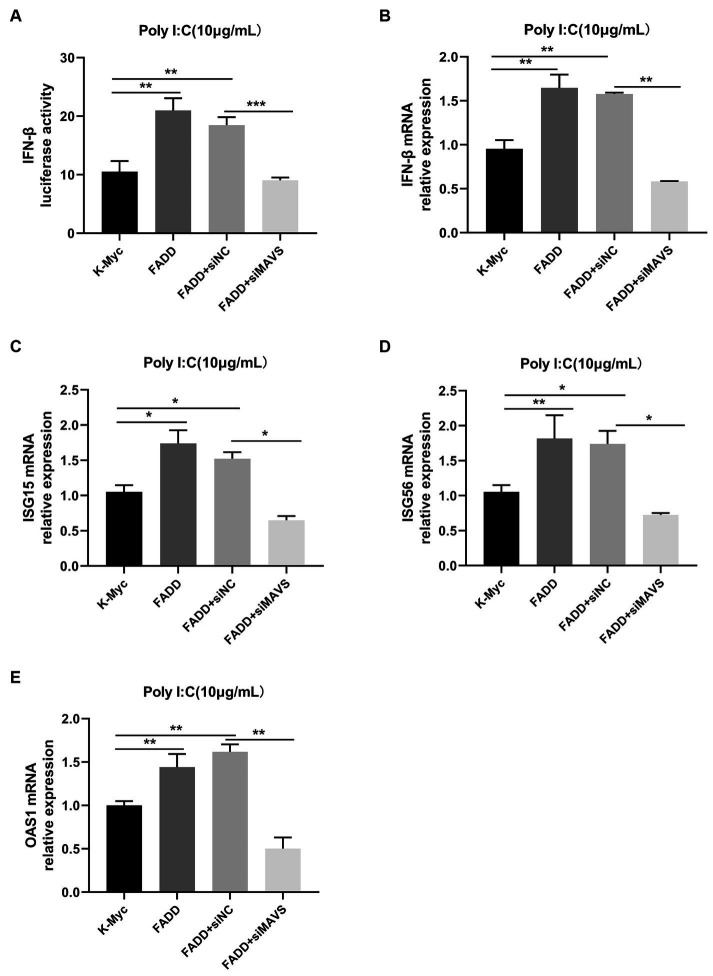
FADD promotes type I IFN signaling in a MAVS-dependent manner. **(A)** MARC-145 cells were transfected with IFN-β reporter plasmid, FADD-Myc, and siNC or siMAVS for 36 h, then the cells were stimulated with polyI:C, and 12 h later, the luciferase activities of IFN-β were assayed by dual-luciferase reporter assay. **(B–E)** MARC-145 cells were transfected with FADD-Myc and siNC or siMAVS for 36 h, and then the cells were stimulated with polyI:C, and 12 h later, the mRNA levels of IFN-β **(B)**, ISG15 **(C)**, ISG56 **(D)** and OAS1 **(E)** were detected by qPCR. The results are representative of three independent experiments (the means ± SD). ^*^p < 0.05, ^**^p < 0.01, and ^***^p < 0.001. The statistical significance of differences was determined using student’s *t*-test.

Since FADD could enhance MAVS-mediated type I IFN signaling, to identify whether MAVS is indispensable for FADD to regulate the production of type I IFN, MARC-145 cells were transfected with siMAVS, FADD-Myc, and IFN-β reporter plasmid, and 36 h later, the cells were stimulated with polyI:C. As shown in Figure 6, FADD promoted polyI:C-induced IFN-β and ISGs (ISG15, ISG56 and OAS1) expressions in siNC cells. Nevertheless, it could not increase polyI:C-mediated production of type I IFN and ISGs upon silencing of MAVS. Collectively, these results suggest that FADD positively regulates MAVS-mediated type I IFN signaling.

### FADD inhibits PRRSV infection via enhancing MAVS-IFN signaling

**Figure 7:**
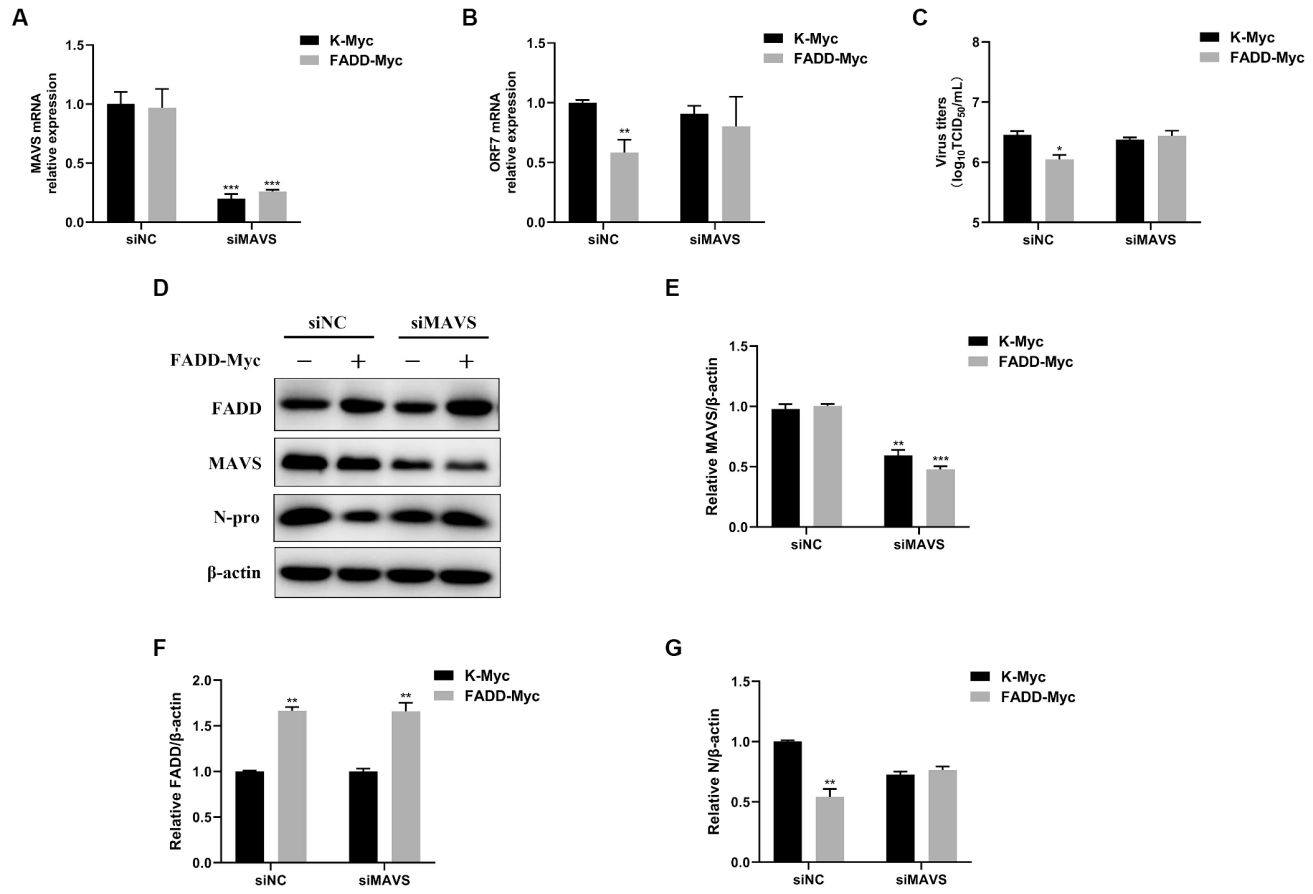
The antiviral activity of FADD is dependent on MAVS. MARC-145 cells were transfected with siMAVS and FADD-Myc, and 24 h later, and the cells were infected with PRRSV at a MOI of 0.1 for 24 h. Then, the cells were harvested, the silencing efficiency of MAVS **(A)** and RNA levels of PRRSV **(B)** were determined by qPCR. **(C)** The virus titers from cell supernatants were analyzed by TCID_50_. **(D)** The expression levels of N protein were detected by western blotting. **(E–G)** The relative intensity ratios of MAVS, FADD and N protein were analyzed using Image J software. ^*^p < 0.05, ^**^p < 0.01, and ^***^p < 0.001. The statistical significance of differences was determined using student’s *t*-test.

In view of the fact that FADD possesses significant antiviral activity and enhances MAVS-mediated type I IFN signaling, to investigate whether the antiviral activity of FADD is dependent on MAVS, MARC-145 cells were transfected with siRNAs of MAVS (siMAVS) and FADD-Myc. As shown in Figure 7, marked reduction of MAVS expression was observed in the MAVS silenced cells (Figures 7A,D,E). And FADD could not decrease the PRRSV RNA levels (Figure 7B) and viral titers (Figure 7C) in the MAVS-silenced cells. Meanwhile, there was no difference in PRRSV N protein between MAVS knockdown cells transfected with K-Myc and FADD-Myc (Figures 7D,F,G). These data indicated that the anti-PRRSV activity of FADD needs the involvement of MAVS.

**Figure 8:**
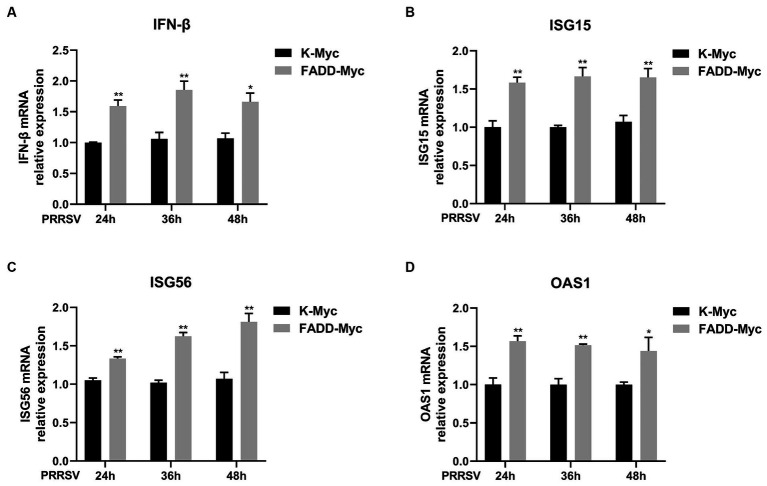
FADD enhances type I IFN signaling during PRRSV infection. MARC-145 cells were transfected with FADD-Myc or control vector for 24 h, and infected with PRRSV at a MOI of 0.1 for another 24, 36 and 48 h. Then, the cells were harvested, and the mRNA levels of IFN-β **(A)**, ISG15 **(B)**, ISG56 **(C)** and OAS1 **(D)** were detected by qPCR. ^*^p < 0.05 and ^**^p < 0.01. The statistical significance of differences was determined using student’s *t*-test.

In addition, we investigated whether FADD regulates the production of type I IFN and ISGs during PRRSV infection. MARC-145 cells were transfected with FADD-Myc or control vector for 24 h, then the cells were infected with PRRSV for 24, 36 and 48 h. Compared to control cells, cells with FADD overexpression significantly enhanced the expression of IFN-β at 24, 36 and 48 h (Figure 8A). Meanwhile, ISG15, ISG56 and OAS1 mRNA expressions were also increased in FADD-overexpressed cells (Figures 8B–D). Overall, FADD promotes anti-PRRSV response through the regulation of IFN-β and interferon-stimulated gene expression mediated by MAVS.

## Discussion

PRRSV has emerged as one of the major threats to global pig industry since its outbreak in the late 1980s (31). Although commercially available vaccinations are widely used, PRRSV is still not effectively controlled due to the antigenic drift. The relationship between PRRSV and its host is complex and delicate. On the one hand, PRRSV has evolved multiple strategies to suppress the production of type I IFNs. On the other hand, innate immunity exerts a key role in response to PRRSV (32, 33). Here, a novel strategy is revealed in which the host protein FADD suppresses PRRSV by positively regulating host innate immune response.

The role of FADD in PRRSV infection was firstly investigated, and the results showed that overexpression of FADD could significantly suppress PRRSV replication (Figure 1), and silencing the expression of endogenous FADD by small interfering RNAs led to the promotion of PRRSV replication (Figure 2). Subsequently, we demonstrated that FADD markedly enhances MAVS-mediated type I IFN signaling (Figure 5). Furthermore, the ability of FADD to antagonize the replication of PRRSV is dependent on MAVS (Figure 7), and during PRRSV infection, FADD can promote the production of type I IFN and ISGs (ISG15, ISG56, and OAS1) (Figure 8). In conclusion, our study uncovered that FADD plays an important role in antiviral immune response.

FADD is an adaptor protein known to be crucial for the mammalian cell extrinsic pathway of apoptosis (34). Apart from its critical role in death receptor signaling of apoptosis, FADD has emerged as a new actor in cancer development, inflammation, innate immunity, and virus infection. It has been reported that FADD^−/−^ MEFs are highly sensitive to infection by many RNA viruses, including influenza virus, VSV and EMCV, due to defective interferon production (21). In this study, we demonstrated that FADD plays an important role in inhibiting PRRSV replication, and it was the first time to give evidence that FADD was a host protein against PRRSV. Besides, many studies have demonstrated that PRRSV induces apoptosis both *in vitro* and *in vivo*. Apoptosis is often considered as an innate defense mechanism that limits virus infection by elimination of infected cells (32). And PRRSV could increase TNFR1/FasL expression levels (35), so maybe FADD has an important role in apoptosis after PRRSV infection. Moreover, we found that FADD inhibition of PRRSV replication depended on MAVS, and FADD could promote the MAVS-mediated production of IFN-β and downstream ISGs, which indicated that MAVS-mediated type I IFN pathway may play a key role in FADD repressing PRRSV replication.

MAVS is a critical adaptor in the RLR signaling pathway that links upstream recognition of viral RNA to downstream signal transduction in the antiviral response (36). It is well known that MAVS aggregation is considered to be a hallmark of its activation and to be essential for its antiviral function. Ubiquitination of RIG-I was shown to be a critical element for it to activate MAVS, RIG-I binds to unanchored lysine-63 (K63) polyubiquitin chains and that this binding is important for MAVS aggregation and activation (37). SNX8 was associated with VISA and increased the aggregation of VISA, leading to the activation of downstream signaling (38). Phosphorylation and dephosphorylation have also been reported to play a critical role in antiviral innate immunity. c-Abl positively regulates the RLR signaling by phosphorylating MAVS at Y9, Y30, and Y71 (39, 40). Besides, protein succinylation caused by succinyl-CoA is a newly discovered novel post-translational modification (41). Sirtuin5 desuccinylates MAVS at K7 to diminish and antagonize the MAVS aggregation and antiviral response (42). Given that the role of FADD in RLRs or MAVS-mediated upstream signaling is rarely explored, further studies on MAVS aggregation and post-translational modifications of MAVS by FADD need to be done to clarify the detailed mechanism of FADD regulating MAVS.

Besides, we also found that FADD could enhance OAS1 expression mediated by RIG-I and TBK1, but did not affect the expression of IFN-β, ISG15 and ISG56. Previous studies have identified that following viral infection, type I IFN signaling induces the production of the OAS family consisting of OAS1, OAS2, OAS3, and OAS-like (OASL) protein. The ubiquitin-like domains of OASL could substitute for K63-linked poly-ubiquitin and interact with the CARDs of RIG-I and thereby enhancing RIG-I signaling. And the crystal structure of the OAS-like domain shows a striking similarity with OAS1 (43, 44), so the roles of FADD in RIG-I-OAS1 signaling axis remains to be further studied.

In summary, our study highlights the importance of FADD in PRRSV replication. We reported that FADD promotes MAVS-mediated type I IFN pathway and the expression of downstream genes. Besides, FADD inhibits PRRSV infection via MAVS-IFN pathway, which contribute to understanding the host antiviral mechanism, and may have implications for the future control of PRRS.

## Data availability statement

The original contributions presented in the study are included in the article/supplementary material, further inquiries can be directed to the corresponding authors.

## Ethics statement

Ethical approval was not required for the studies on humans in accordance with the local legislation and institutional requirements because only commercially available established cell lines were used. Ethical approval was not required for the studies on animals in accordance with the local legislation and institutional requirements because only commercially available established cell lines were used.

## Author contributions

XC: Data curation, Formal analysis, Methodology, Writing – review & editing, Writing – original draft. MW: Data curation, Formal analysis, Writing – original draft. ZL: Data curation, Formal analysis, Writing – original draft. LW: Formal analysis, Methodology, Writing – original draft. GZ: Supervision, Writing – original draft. YC: Supervision, Writing – original draft. JH: Supervision, Writing – original draft.
